# Sustained synchronized neuronal network activity in a human astrocyte co-culture system

**DOI:** 10.1038/srep36529

**Published:** 2016-11-07

**Authors:** Jacobine Kuijlaars, Tutu Oyelami, Annick Diels, Jutta Rohrbacher, Sofie Versweyveld, Giulia Meneghello, Marianne Tuefferd, Peter Verstraelen, Jan R. Detrez, Marlies Verschuuren, Winnok H. De Vos, Theo Meert, Pieter J. Peeters, Miroslav Cik, Rony Nuydens, Bert Brône, An Verheyen

**Affiliations:** 1 Hasselt University, Biomedical Research Institute, Diepenbeek, B-3590, Belgium; 2 Janssen Research & Development, a division of Janssen Pharmaceutica N.V, Beerse, B-2340, Belgium; 3 Antwerp University, Department of Veterinary Science, Antwerp, B-2020, Belgium; 4 Ghent University, Department of Molecular Biotechnology, Ghent, B-9000, Belgium

## Abstract

Impaired neuronal network function is a hallmark of neurodevelopmental and neurodegenerative disorders such as autism, schizophrenia, and Alzheimer’s disease and is typically studied using genetically modified cellular and animal models. Weak predictive capacity and poor translational value of these models urge for better human derived *in vitro* models. The implementation of human induced pluripotent stem cells (hiPSCs) allows studying pathologies in differentiated disease-relevant and patient-derived neuronal cells. However, the differentiation process and growth conditions of hiPSC-derived neurons are non-trivial. In order to study neuronal network formation and (mal)function in a fully humanized system, we have established an *in vitro* co-culture model of hiPSC-derived cortical neurons and human primary astrocytes that recapitulates neuronal network synchronization and connectivity within three to four weeks after final plating. Live cell calcium imaging, electrophysiology and high content image analyses revealed an increased maturation of network functionality and synchronicity over time for co-cultures compared to neuronal monocultures. The cells express GABAergic and glutamatergic markers and respond to inhibitors of both neurotransmitter pathways in a functional assay. The combination of this co-culture model with quantitative imaging of network morphofunction is amenable to high throughput screening for lead discovery and drug optimization for neurological diseases.

Neurons form connections driven by molecular pathways that are encoded by developmental programs. Refinement of this neuronal network highly depends on spontaneous and experience-driven electrical activity stimulating synaptic connectivity and maturation^1^,^2^,^3^. As a result, spontaneous neuronal activity, most often exemplified by intracellular calcium bursting behavior, synchronizes during central nervous system development to form robust neuronal network activity via synaptic contacts. Highly synchronized bursting has been observed in different brain regions *in vivo*^2^,^4^, but also *in vitro* in brain slices^5^,^6^, and even in dissociated primary neuronal cultures^1^,^7^. Therefore, spontaneous activity is thought to be an intrinsic property of neurons, regulating synaptic transmission efficacy and cytoplasmic protein and membrane receptor trafficking^2^,^3^,^8^.

Various neurodevelopmental and neurodegenerative disorders are associated with cognitive deficits. A characteristic trait of these disorders is that the morphofunction (i.e. structural and functional differences) of neuronal networks underlying cognition becomes compromised^9^ (reviewed in ref. 10). For example, synaptic degeneration, network remodeling, and abnormal synchronization of neuronal network activity are underlying cognitive deficits in Alzheimer’s disease^11^,^12^. In epilepsy increasing neuronal excitability and hypersynchrony disrupt normal brain function^13^,^14^, while hyposynchrony during development is suggested to underlie the pathology observed in schizophrenia patients^15^.

Sensitive assays have been developed for measuring morphofunctional connectivity of neuronal networks, including synchronized calcium bursting behavior in primary cultures of rodent neurons^7^,^16^,^17^. Such assays have been exploited to assess various pharmacological and genetic interventions^18^. Additionally electrophysiology studies *in vivo* as well as on acute brain slices have shown their importance, as they mimic aspects of particular brain areas and the (patho-) physiologically developed wiring of these structures. However, animal models often fail to mimic all features of human disease and to date translational value remains poor^19^,^20^, potentially due to species-specific features of particularly those brain structures like the cerebral cortex that are thought to be essential for human-specific cognitive functions (reviewed in ref. 21). Therefore, fully human-derived models would be extremely valuable for studying disease mechanisms and identifying new therapeutic targets for neurodevelopmental and neurodegenerative diseases. The discovery of human induced pluripotent stem cells (hiPSCs)^22^ has enabled the study of genetic diseases in a lineage-specific context using patient-derived cells^23^. Human-derived models facilitate a better understanding of complex genetic disorders or polygenic diseases in contrast to most animal models which are often artificially mimicking only some aspects of a certain disorder^24^. They provide a valuable addition to cells derived from animal models, which are currently the mainstay for disease modeling and drug discovery. Patient-derived cells can also serve as powerful tool for identification of new therapeutic targets and optimization of drug treatments in personalized medicine^24^.

Experimental models using hiPSC-derived neurons could be of particular relevance for neurodevelopmental and neurodegenerative disorders with complex etiologies like autism, schizophrenia, and Alzheimer’s disease^25^,^26^, as the complex genetic background is difficult to mimic using mutant animal models. However, the currently available protocols to study robust functional network activity and connectivity in hiPSC-derived cortical neurons are often time-consuming and highly variable^20^,^27^,^28^,^29^,^30^.

Functional maturation of human neurons has been shown to be improved when co-cultured with rodent astrocytes^29^,^31^,^32^. However, rodent astrocytes significantly differ from human astrocytes^33^,^34^ and a co-culture model of human iPSC-derived cortical neurons with human primary astrocytes is currently not described to our knowledge. This co-culture model could be further exploited to study functional interactions between patient iPSC-derived astrocytes and neurons.

In this study we describe the morphofunctional characterization of a fully human iPSC-derived neuron-astrocyte co-culture model. We show that optimal co-culture conditions allow the formation of synchronized neuronal network activity, within a timespan of four weeks after final plating. These neurons express both GABAergic and glutamatergic markers and respond to inhibitors of both neurotransmitter pathways. Passaging and upscaling of neural precursor cells before final plating decreased the ability to form functional neuronal networks. We present a robust and convenient protocol based on 96 multi-well format to obtain functionally connected networks of hiPSC-derived cortical neurons demonstrating sustained synchronized network activity.

## Results

### Differentiated hiPSC-derived cortical neurons associate in dense clusters on laminin coated surfaces

**Figure 1:**
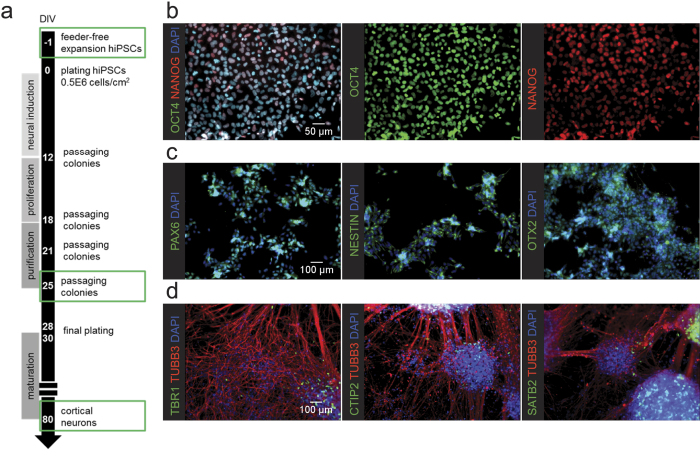
Characterization of hiPSC (-derived) cells during different steps of the differentiation protocol on laminin coated surface (**a**) Schematic overview of the differentiation protocol towards hiPSC-derived cortical neuronal cultures. (**b**) hiPSCs express pluripotency markers OCT4 and NANOG before the start of differentiation. (**c**) Neural precursor cells express neural stem cell markers PAX6, nestin, and OTX2 at the 25^th^ day of the differentiation protocol. (**d**) Fully differentiated neurons express neuronal marker class III β-tubulin and cortical markers TBR1, CTIP2, and SATB2. However, due to the heterogeneous nature of the cultures, not all cells are immuno-positive for all markers (also undifferentiated neural precursor cells and potentially some astrocytes are present in the cultures).

In order to study neuronal function and connectivity *in vitro*, we adapted a previously established cortical differentiation protocol (Fig. 1a)^25^ for differentiating hiPSCs into cerebral cortex neurons. Starting from commercially available hiPSCs (Sigma or Cellectis) (Fig. 1b) neural differentiation was induced. Neural progenitor phenotype was confirmed by PAX6, Nestin and OTX2 staining (Fig. 1c) at 25 days after starting neural induction. After proliferation and purification, neural precursor cells (NPCs) were cryopreserved until further use. Final plating of NPCs in neural maintenance medium on a laminin-coated surface further differentiated cells into cortical neurons expressing neuronal marker class III β-tubulin and cortical markers TBR1, CTIP2, and SATB2 (Fig. 1d) at 80 days after neural induction (7 weeks after final plating). However, also non-differentiated progenitor cells were still present in the cultures. Furthermore, the cells clustered and detached easily (Fig. 1d), thereby complicating downstream analyses.

### Astrocyte co-cultures and Notch signaling inhibition increase the homogeneity of hiPSC-derived cortical neurons

**Figure 2:**
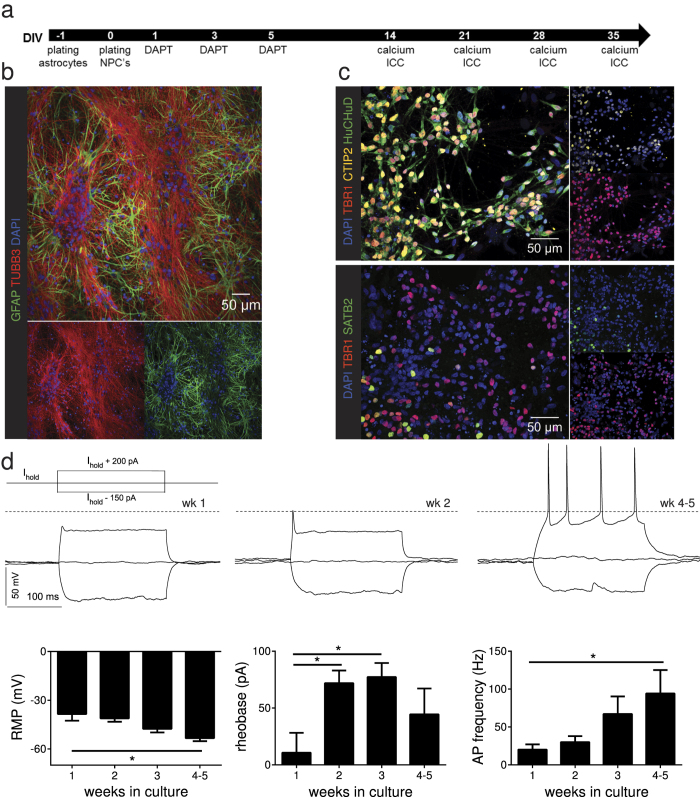
Functional maturation of hiPSC derived cortical neurons in a short time frame via co-culture with astrocytes and treatment with DAPT (**a**) Cortical neurons are differentiated from NPCs after final plating on top of an astrocyte monolayer. During the first week of differentiation DAPT is added. Functional and morphological assays are performed at 2–8 weeks after final plating. (**b**) hiPSCs differentiated towards cortical neurons in co-culture with primary human astrocytes are stained with neuronal marker class III β-tubulin, astrocyte marker GFAP and nuclear marker DAPI. (**c**) Cortical fate of hiPSC-derived neurons grown in human astrocyte co-cultures is confirmed by immunocytochemistry for cortical markers TBR1, CTIP2, and SATB2 in combination with the nuclear marker DAPI. (**d**) Functional maturation of neurons analyzed using whole cell patch clamp. Traces of evoked potentials (protocol scheme included) show a clear difference between early (wk1 and wk2 respectively firing no action potentials or only one) and later time points (wk4-5 firing repetitive action potentials) of differentiation. The left graph shows a more negative resting membrane potential over time (One-way ANOVA, p = 0.0113). The middle graph shows an increasing maximum action potential frequency over time (One-way ANOVA, p = 0.0187). An increased rheobase is observed as well two and three weeks after final plating (One-way ANOVA, p = 0.0112). n ≥ 9 from ≥1 differentiation, mean + SEM, *p < 0.05.

Different research groups have already shown that rodent astrocytes improve the maturation of hiPSC-derived neurons^29^,^31^. Hence, in order to reduce cell clustering and detachment of hiPSC-derived cortical neuronal networks, we established co-cultures with primary human fetal astrocytes provided by ScienCell™. Immunocytochemistry and microarray data (Supplementary Fig. S1) on different passage numbers of these human primary astrocytes revealed that the expression of astrocyte markers GFAP and S100B (mRNA and protein) decreased with increasing passage numbers. Therefore, only astrocytes passaged for a maximum of 3 times were used for co-cultures. These neuron/astrocyte co-cultures effectively reduced neuronal clumping and detachment *in vitro* (Fig. 2b).

Since studies of stem cell derived neuronal cultures can be impeded by ongoing progenitor proliferation and neurogenesis creating a culture with heterogeneous neuronal identities, a Notch signaling inhibitor (N-[N-(3,5-Difluorophenacetyl)-L-alanyl]-S-phenylglycine t-butyl ester, DAPT) was used to synchronize the cultures by stimulating differentiation and inhibiting proliferation of all NPCs at the same time^35^,^36^.

In order to confirm preservation of cortical fate of the neuronal co-cultures, immunocytochemical stainings for cortical markers TBR1, CTIP2, and SATB2 were performed (Fig. 2c). Three weeks after final plating the cultures contain–next to the added astrocytes and undifferentiated NPCs–about 60% neurons (HuCHuD positive nuclei 62% ± 11%), of which 38% (±6%) were TBR1 positive, 41% (±3%) CTIP2 positive, and 12% (±4%) SATB2 positive, suggesting that early generated deep layer neurons were predominantly present.

### Functional maturation of hiPSC-derived neurons co-cultured with human astrocytes

Electrophysiological recordings were performed to test functionality and maturity of hiPSC-derived cortical neurons in co-cultures treated with DAPT. Whole-cell patch clamp recordings showed a gradual maturation of single cells within the network over time (Fig. 2d), as evident by a more negative resting membrane potential (p = 0.0113). In support of the progressive maturation, an increase in rheobase was observed^37^ (i.e. the minimal current sufficient to induce an action potential) at the second and third week after final plating (p = 0.0112). The number of neurons firing action potentials (wk1: 52%, wk2: 55%, wk3: 64%, wk4–5: 100% of all neurons recorded) and the respective firing frequency also increased over time (p = 0.0187) confirming maturation of cortical neurons^37^.

### Human neuron/astrocyte co-culture in combination with simultaneous differentiation of NPCs improves synchronization of calcium oscillations and network activity

**Figure 3:**
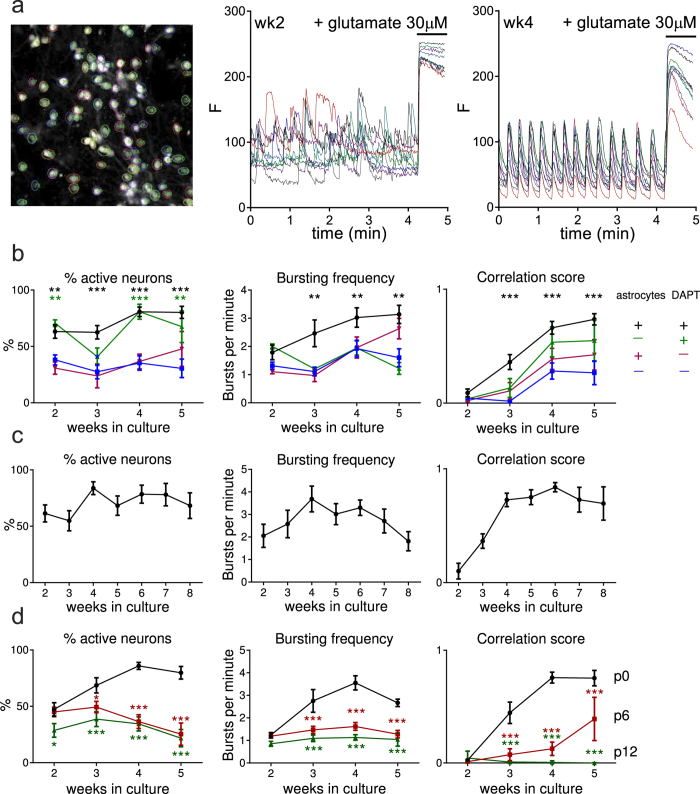
Optimization of conditions for synchronized neuronal calcium oscillations (**a**) Image showing automated identification of FLUO-4 loaded cells by color-coded regions of interest (ROIs) and representative traces (each trace represents fluorescence of one cell) of co-cultures with DAPT two weeks after final plating (left, no synchronicity) and four weeks after final plating (right, highly synchronized calcium influxes). After 250 frames (61 frames per minute) 30 μM glutamate was added, resulting in a large calcium influx and used to distinguish neurons from astrocytes. (**b**) Co-culturing with primary human astrocytes and treatment with DAPT significantly increases the percentage of active neurons (Two-way ANOVA, p < 0.0001), bursting frequency (Two-way ANOVA, p < 0.0001) and synchronicity (Two-way ANOVA, p < 0.0001) compared to cultures without DAPT and astrocytes. n ≥ 4 from ≥2 differentiations, mean ± SEM, **p < 0.01,***p ≤ 0.0001. (**c**) Synchronized activity sustains up to 8 weeks after final plating in human astrocyte co-cultures with DAPT. n ≥ 5 from ≥2 differentiations, mean ± SEM. d) Limited FGF2 passaging significantly reduces the percentage of active neurons (Two-way ANOVA, p < 0.0001), bursting frequency (Two-way ANOVA, p < 0.0001) and synchronicity (Two-way ANOVA, p < 0.0001). n ≥ 4 from ≥2 differentiations, mean ± SEM, *p < 0.05, ***p ≤ 0.0001.

In addition to electrophysiological recordings on single cells we studied the development of neuronal connectivity on neuronal network level with live cell calcium imaging. Using an in-house optimized assay (Fig. 3a)^16^, functionality of differentiating hiPSC-derived neuronal networks was followed over a time course of 2–5 weeks after final plating. Neural precursor cells were plated with or without human astrocytes in the presence or absence of DAPT (Fig. 3b). We focused on the percentage of active neurons, bursting frequency and synchronicity (i.e. the mean Pearson’s correlation between calcium indicator traces of single cells^16^). All conditions were compared to the initial protocol, which is monoculture without DAPT (Fig. 3b). When neurons were co-cultured with primary human astrocytes in the presence of DAPT, the percentage of active neurons was significantly increased at all time points measured while bursting frequency and synchronicity were significantly higher from week 3 onwards compared to monocultures (Fig. 3b). All measured parameters reached a plateau 4 to 5 weeks after final plating and remained high at least up to week 8 (Fig. 3c). The individual contributions of DAPT treatment and astrocyte co-culture were questioned further. Co-culture with astrocytes (without DAPT) did not improve any of the parameters, while DAPT treatment (without astrocytes) only increased the amount of active neurons. Therefore, the optimal condition is co-culturing with DAPT.

In order to compare fully human co-cultures with mixed rodent/human co-cultures (as previously described^29^,^31^), we cultured our hiPSC-derived neurons with freshly dissected rat primary astrocytes in the presence of DAPT. Mixed rat/human co-cultures showed an increased percentage of active neurons, bursting frequency and synchronization of neuronal calcium oscillations (Supplementary Fig. S2) 2 to 5 weeks after final plating compared to monocultures, which is much faster than in previous studies (>2 months)^29^,^31^. Additionally, there is a significantly increased percentage of active neurons, bursting frequency and correlation score in mixed co-cultures compared to fully human co-cultures at the earliest time points (wk 2 and 3), while the percentage of active neurons decreased significantly at the latest time point measured (wk5). Nevertheless, with our aim to generate a fully human model, we only focus on human astrocytes for all further co-culture experiments.

### Passaging and upscaling of NPCs before final plating decreases network activity

Increasing the amount of FGF2 passages of neural precursor cells has been shown to increase the yield of the neural induction^38^. Hence we explored the possibility of using higher passage numbers of NPCs by a limited amount of FGF2 passages (6 or 12 passages, passaging twice a week) before cryopreservation and final plating. Notably, live cell calcium imaging showed that even a limited amount of FGF2 passages (for 6 or 12 times) decreased neuronal network functionality (Fig. 3d), lowering the percentage of active neurons (p < 0.0001), bursting frequency (p < 0.0001) and synchronicity (p < 0.0001). Therefore, FGF2 passaging is not recommended and we did not use passaged NPCs for other experiments in this study.

### Synchronized calcium oscillations likely represent neuronal network activity

**Figure 4:**
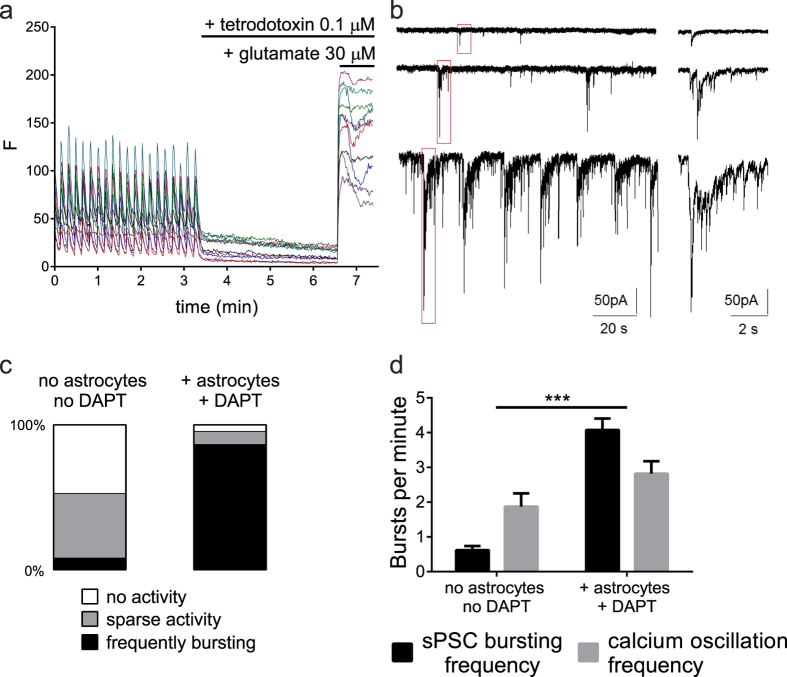
Synchronized calcium oscillations represent neuronal network activity (**a**) Representative traces of live cell calcium imaging recordings in 5 week old cortical neuronal co-cultures, FLUO-4 intensity is shown over time (61 frames per minute). After 200 frames cells are exposed to tetrodotoxin (0.1 μM) followed by addition of glutamate (30 μM) after 200 frames, resulting in a large calcium influx. (**b**) Representative traces of patch clamp recordings of sPSCs showing sparse sPSCs (upper trace), sparse bursts of sPSCs (middle trace) and frequent bursting (lower trace). (**c**) Co-culture with primary human astrocytes + DAPT increases the percentage of cells with sPSC bursts compared to control (no DAPT, no astrocytes). “No activity” represents up to five single sPCSs per minute, “sparse activity” reflects 5 or more single events or less than one burst per minute and cells bursting at a frequency higher than one burst per minute are labeled with “frequently bursting”. Number of cells per cell culture condition n ≥ 22, from ≥2 differentiations. (**d**) Frequency of sPSC bursts per minute in co-cultures with primary human astrocytes + DAPT or neuron-only cultures equals the calcium oscillation frequency (Two-way ANOVA, p = NS for the respective cell culture conditions), while culture conditions induce significantly different bursting frequencies (Two-way ANOVA, p ≤ 0.0001). sPSC frequency: number of patched cells per cell culture condition ≥ 22, from ≥2 differentiations, calcium oscillation frequency: n ≥ 6 from ≥2 differentiations. Mean + SEM, ***p ≤ 0.0001.

In order to explore the nature of the recorded calcium oscillations, we performed additional experiments. First, treatment of co-cultures with tetrodotoxin (TTX), an inhibitor of voltage-gated sodium channels, completely blocked all calcium oscillations (Fig. 4a), suggesting that calcium oscillations are a secondary effect to voltage-gated sodium channel mediated action potentials. Moreover, this observation excludes astrocyte-induced calcium oscillations in our model^39^. Furthermore, we measured spontaneous postsynaptic currents (sPSCs, Fig. 4b) in co-cultures compared to monocultures. The spontaneous electrical activity represented synaptic events because the burst firing was completely abolished in 5 out of 7 cells by combined application of the competitive AMPA/kainate receptor antagonist CNQX (20 μM) and competitive NMDA receptor antagonist DAP5 (50 μM) in the recording buffer during 5 minutes (data not shown). Co-cultures displayed more bursts of postsynaptic events (86.4% of cells) than monocultures (7.9% of cells) 5 weeks after final plating (Fig. 4c). More specifically, monocultures were mainly inactive (47.4% of cells compared to only 4.5% in co-cultures) or only showed sparse activity (44.7% of cells compared to 9.1% in co-cultures). These patch clamp experiments confirm that bursts of activity increase as cultures become more mature^40^. Finally, the frequency of sPSC bursts is similar to the frequency of calcium oscillations in both conditions (Fig. 4d), while there is a clear difference in frequency between the two cell culture conditions (Fig. 4d). These findings strongly suggest that synaptic network activity underlies the synchronicity of the calcium oscillations in the iPSC-derived neuronal co-culture network.

### GABAergic and glutamatergic contribution to network function in human astrocyte co-cultures

A balanced contribution of GABAergic and glutamatergic transmission to neuronal activity has been shown to be crucial for proper network function. Presence of sufficient glutamatergic neurons seems to be critical for maturity and function of a network^41^. However, GABAergic signaling has been shown to be as important for maturation and network development^42^. Therefore, the contribution of GABAergic and glutamatergic transmission to the neuronal activity in our co-cultures was studied using live cell calcium imaging. GABAergic and glutamatergic neurotransmission was studied at 3, 5, and 7 weeks after final plating by inhibiting their respective contributions. Responses were analyzed after recording baseline activity for 200 frames (61 frames per minute) by acute addition of respectively GABA_A_ receptor inhibitor picrotoxin (50 μM) into the recording buffer or the combination of NMDA and AMPA receptor blockage with CNQX (20 μM) and DAP5 (50 μM) into the recording buffer. The effect of glutamatergic and GABAergic inhibition was recorded for another 200 frames.

**Figure 5:**
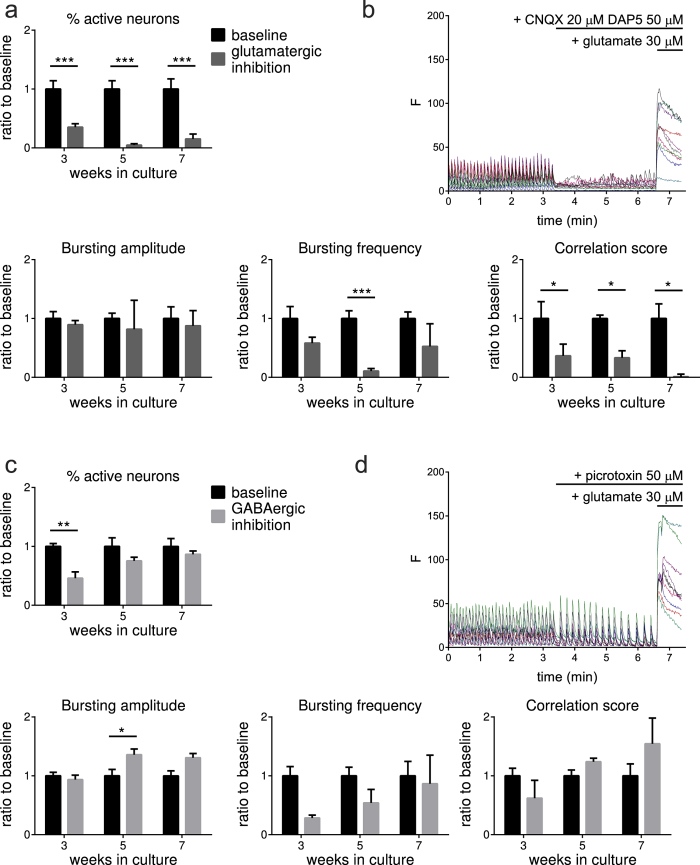
GABAergic and glutamatergic contribution to neuronal network function for cortical neurons in co-culture (**a**) Graphs showing a clear contribution of glutamatergic transmission to the calcium signal. Percentage of active neurons (One-way ANOVA, p < 0.0001), frequency (One-way ANOVA, p = 0.0002) and synchronicity (One-way ANOVA, p = 0.0004) significantly change compared to baseline. Mean + SEM, n ≥ 3, from 3 differentiations. *p < 0.05, ***p ≤ 0.0001 on the different time points. (**b**) Representative traces of live cell calcium imaging recordings in 5 week old cortical neuronal co-cultures, FLUO-4 intensity is shown over time (61 frames per minute). After 200 frames cells are exposed to DAP5 (50 μM) and CNQX (20 μM) followed by exposure to glutamate (30 μM) after 200 frames, resulting in a large calcium influx. (**c**) Graphs showing the contribution of GABAergic transmission to the calcium signal. The percentage of active neurons (One-way ANOVA, p = 0.0015), bursting frequency (One-way ANOVA, p = 0.0302) and burst amplitude (One-way ANOVA, p = 0.0125) are significantly changed after addition of picrotoxin. Mean + SEM, n ≥ 3, from 3 differentiations. *p < 0.05, **p < 0.01 on the different time points (**d**) Representative traces of calcium imaging. After 200 frames, the cells are exposed to the GABAergic inhibitor picrotoxin (50 μM), followed by glutamate exposure (30 μM) after 200 frames.

Glutamatergic inhibition of network activity significantly reduced the percentage of active neurons (p < 0.0001 for all time points) and their synchronicity (p < 0.05 for all time points). The bursting frequency was reduced as well, although only at week 5 (p < 0.001) while the bursting amplitude remained unaffected on all time points (p = NS) (Fig. 5a). Representative traces are shown in Fig. 5b.

On the other hand, GABAergic inhibition significantly reduced the percentage of active neurons (p = 0.0015), but the decrease was only significant at week 3 (p < 0.01). Synchronicity was unaffected (p = NS). Addition of picrotoxin also affected the bursting frequency (overall p = 0.0302) and the bursting amplitude (overall p = 0.0125) with a significant increase at week 5 (p < 0.05) (Fig. 5c). Representative traces are shown in Fig. 5d.

### Human astrocyte co-cultures show expression of both GABAergic and glutamatergic vesicular proteins

**Figure 6:**
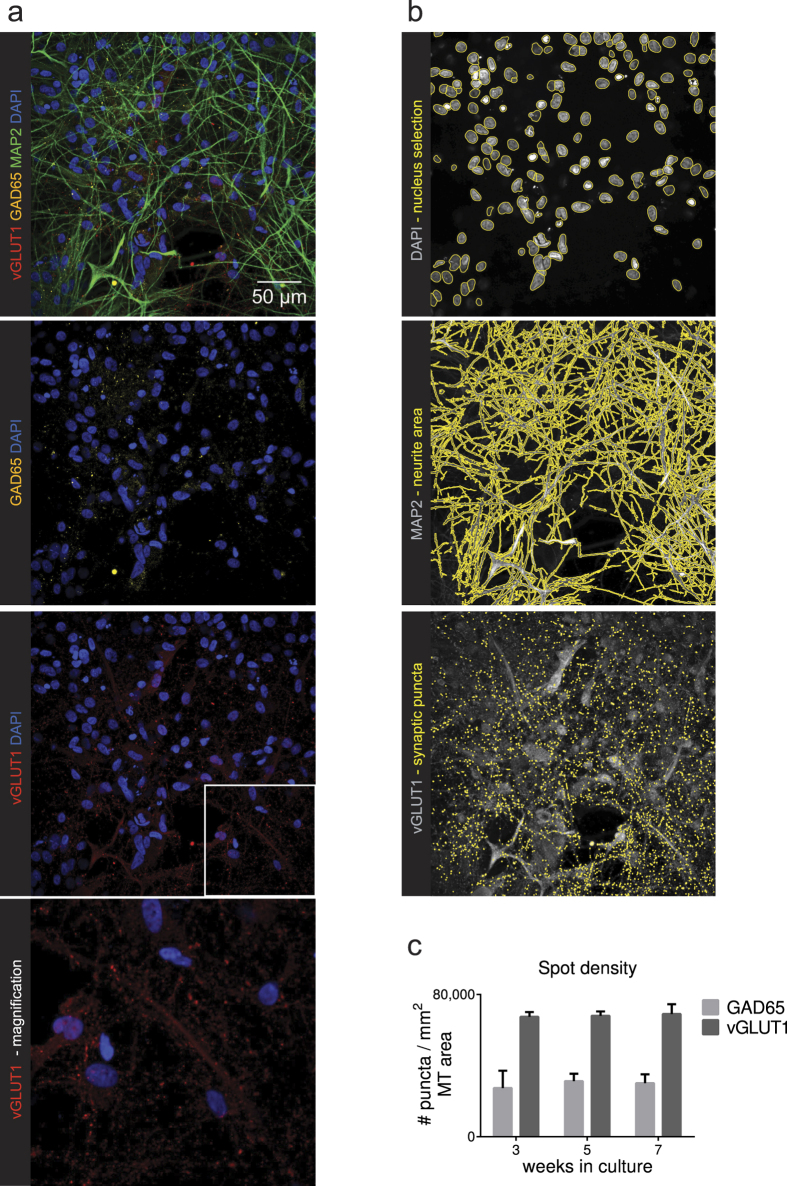
Astrocyte co-cultures are suitable for HCI and analyses (**a**) hiPSCs differentiated towards cortical neurons in co-culture with primary human astrocytes show expression of glutamatergic marker vGLUT1, GABAergic marker GAD65, and neuronal marker MAP2. (**b**) Image analysis based on raw data files from a plate scanner. Masks (in yellow) are drawn per channel to identify the number of nuclei (based on DAPI staining), neurite area (based on MAP2 staining) and the number of puncta per marker (either based on vGLUT1 or GAD65). (**c**) Quantification of glutamatergic marker vGLUT1 and GABAergic marker GAD65 per neurite area in human astrocyte co-cultures. Mean + SEM, n ≥ 4, from 3 differentiations.

To verify whether the functional activity is also reflected by morphological correlates of connectivity we performed an automated analysis of synaptic marker expression in hiPSC-derived cortical neurons (Fig. 6a,b). Expression of glutamatergic and GABAergic vesicular proteins vGLUT1 and GAD65 complemented the contribution of both neurotransmitter pathways to the observed neuronal network function (Fig. 6c). At all three time points, similar levels of vGLUT1 and similar levels of GAD65 expression per neurite surface were observed.

## Discussion

In the present study we report a fully human iPSC-derived cortical neuron/primary astrocyte co-culture system with sustained synchronized network activity. We found that differentiating hiPSC-derived neuronal cells in co-cultures progressively displayed more negative resting membrane potentials to a level sufficient to remove voltage-dependent inactivation of sodium channels and to fire repetitive action potentials. An increasing percentage of hiPSC-derived neurons fired action potentials over time, consistent with the extensive electrophysiological characterization of hiPSC-derived neurons that was published previously^43^. Besides the more negative resting membrane potential, a maturation-induced increase of the sodium channel density could contribute to the gradual increase in action potential firing capacity of the hiPSC-derived neurons. Moreover, simultaneously differentiated neurons in a human astrocyte co-culture system revealed increased activity, bursting frequency and calcium oscillation synchronization over time, representative of neuronal network activity. Human iPSC-derived neurons expressed cortical markers TBR1, CTIP2, and SATB2 indicative of both deep and upper layer cortical neurons and reached a purity of about 60% of the total cell population.

Development of the central nervous system is characterized by the occurrence of spontaneous synchronized neuronal activity^8^. To study cortical network connectivity, synchrony, and function, mostly rodent brain slices and primary cortical neuronal cell cultures are used. For example, *ex vivo* population synchrony was found in hippocampal and cortical slices^5^,^6^, and synchronized bursts were recapitulated using dissociated primary neuronal cultures *in vitro*^1^,^7^. More recently, hiPSC-derived cortical neurons have been shown to display bursts of synchronized network activity as well, within a specified time span of differentiation^30^. We were not able to reproduce these data, which might be due to subtle differences in the differentiation protocol, usage of different hiPSC lines, reprogramming methods or methods to measure spontaneous calcium activity. When co-culturing our hiPSC-derived neurons with primary human astrocytes in the presence of the Notch signaling inhibitor DAPT the most reproducible and sustained synchronized network activity was achieved, starting three weeks after final plating of neurons (~DIV50) and sustainable up to 8 weeks (~DIV90) *in vitro,* with neglectable variation between two different hiPSC lines. The support of primary human astrocytes, via released growth factors and physical contact^31^, in combination with forced (DAPT) simultaneous differentiation of NPCs into neurons probably leads to more homogenous and mature networks in a shorter time frame. This way, there is no need to wait for the delayed emergence and development of astrocytes from NPCs, which causes slow but progressive maturation of the network^44^.

Furthermore, we were able to obtain mature networks using rodent astrocytes in about three weeks after final plating, which is much faster than in previously published work (2 months after final plating)^29^,^31^. This difference can result from differences in hiPSC lines, passage numbers before final plating, neural differentiation protocols or methods to measure network maturation. Although synchronization of calcium oscillations appeared earlier in mixed co-cultures than in fully human co-cultures, the percentage of active neurons decreased over time reaching levels of monocultures at week 5. This difference might be due to a “species-specific ‘clock’” regulating neuronal maturation for different species with different kinetics^21^. Rodent astrocytes potentially have faster kinetics than human astrocytes. However, with our aim to generate a fully human model, we only focused on human astrocyte co-cultures.

We confirm with our model that the balance between glutamatergic and GABAergic neurons plays an important role in the observed network activity. This might explain why it is challenging to obtain synchronized oscillations^32^,^41^. During early cortical development, GABA is predominantly excitatory^45^, followed by a switch to inhibitory properties during further maturation^46^,^47^. This supports our observations that both glutamatergic and GABAergic inhibitors reduce the amount of active neurons, bursting frequency and synchronicity at three weeks post final plating while at later time points (5–7 weeks) only inhibition of excitatory glutamatergic activity seems to affect network activity, while protein expression levels do not change over time. Notably, a trend towards increased synchronicity is observed after addition of GABAergic inhibitor picrotoxin at later time points suggesting that inhibitory neurons play an important role in controlling network activity (reviewed in ref. 48). As shown in primary rodent cortical neurons before, burst amplitude was significantly increased at later time points by picrotoxin as well^49^. This is another important difference to the study conducted by Kirwan and colleagues^30^, who were unable to show an effect of GABAergic inhibition on calcium oscillations.

Although further optimization and upscaling is necessary the presented neuron-astrocyte co-culture model could be used to screen for compounds that affect network functionality, either acute or chronically, in the context of phenotypic neurotoxicity screening. Due to the high sensitivity of the calcium assay, subtle changes in network activity might be detected, while other readouts like neurite outgrowth are still unaffected^7^. These combined readouts, in high-content format, allow for gauging neuronal networks. Furthermore, the model might offer a valuable tool for preclinical research and to screen for compounds that restore proper network functionality and reduce the progression of neuronal pathologies that display disturbed network activity and connectivity. For example, the potential mechanisms underlying the lack of neural synchronicity observed in schizophrenia^15^ could be explored. Increased bursting frequency and synchronized oscillations as seen in Parkinson’s disease^50^, FTD^51^,^52^ and epilepsy^11^,^13^,^14^ and more complex dysregulation of network function as reported in Alzheimer’s disease^11^,^53^ and autism (reviewed in ref. 10) could be studied as well.

However, future research will have to demonstrate if a fully human co-culture system can be used with patient iPSC-derived astrocytes and neurons and whether impaired functional networks can be reversed using compounds or gene editing technologies. Furthermore, the interaction between human astrocytes and neurons could be studied by culturing diseased astrocytes with healthy neurons or vice versa. Finally, by adding human iPSC-derived or primary microglia, a triple co-culture model could eventually be a tool to study neuro-inflammation.

In summary, we report that simultaneously differentiated hiPSC-derived cortical neurons in co-culture with human primary astrocytes show increasing maturation of network functionality and synchronicity over time when compared to cultures without primary human astrocytes. Our approach is exquisitely suited for sensitive high-content screening approaches such as neurotoxicity screening, target identification and validation, disease modeling, and phenotypic drug screening potentially leading to safer and more efficacious medicines.

## Methods

### Cell culture conditions

All work with human derived cells was done in accordance with the Belgian guidelines and regulations and informed consent was obtained from the subjects according to manufacturers. hiPSC lines ChiPSC6b_m1 (Cellectis) and iPSC0028 (Sigma) derived from healthy individuals were cultured in Matrigel™ (BD Biosciences) coated 6 multiwell (MW6) plates (Nunc) in mTeSR™1 medium (Stem Cell Technologies) and passaged with EDTA (Gibco) when confluent^54^. hiPSCs were upscaled by passaging for a maximum of 6 times and cryopreserved until further use. Pluripotency was checked before the start of differentiation.

For differentiation into neural precursor cells (NPCs) an adapted version of the protocol from Shi and colleagues^25^ was used. Briefly, confluent hiPSCs were Accutase passaged and plated at 500,000 cells/cm^2^ on Matrigel™ coated MW6 plates in mTeSR™1 complete medium supplemented with 10 μM ROCK inhibitor for 1 day (days *in vitro* (DIV) -2). The day after medium was changed completely with mTeSR™1 complete medium (DIV -1). At DIV0, neural induction was started by changing the medium into N2B27 (Neurobasal medium and DMEM:F12 Glutamax medium in a ratio 1:1, 1% B27 supplement, 1 mM Glutamax, 0.5x Pen/Strep, 0.5% N2 supplement, 2.5 μg/ml insulin (Sigma), 50 μM 2-mercaptoethanol, 0.5x MEM NEAA, 500 μM sodium pyruvate (all Thermo Fisher Scientific, unless stated otherwise) supplemented with 10 μM SB431542 (Sigma), and 1 μM dorsomorphin (Tocris Bioscience) for a total of 12 days. At DIV 12, the neuroepithelial sheet was mechanically broken into large aggregates and replated onto laminin (Sigma) coated MW6 plates. At DIV 13 and 15 medium was replaced with N2B27 medium supplemented with 20 ng/ml FGF2 (Stem Cell Technologies) for neural precursor cell (NPC) expansion. Neural rosettes were purified on DIV 18 using dispase (Sigma, 10 mg/ml in PBS, 1/10 diluted in medium). Large rosette clumps were maintained for another week with 1 or 2 more dispase passages depending on purity of the rosettes. Around day 25 of neural induction, cells were passaged with accutase (Thermo Fisher Scientific) to dissociate cell clumps into a single-cell suspension followed by plating on laminin-coated MW6 plates with N2B27 medium changes every other day. NPCs (DIV 27–30, passage 0) were cryopreserved, further proliferated/passaged in N2B27 medium with 10 ng/ml FGF2, or used for final plating and differentiation into cortical neurons.

Final plating was done between DIV 28 and 31 of differentiation on poly-L-ornithine (PLO) plus laminin (10 μg/ml, both Sigma) coated multiwell plates or coverslips with or without primary human astrocytes (1:4 ratio astrocytes to NPCs). Since different batches of laminin from two suppliers (Biolamina and Sigma) seemed to cause a lot of variation in the attachment and balance between proliferation and differentiation of cells, we only used laminin from Sigma (three different batches) for all experiments described in this study.

To exit the cell cycle for all neurons at the same time point, N-[N-(3,5-Difluorophenacetyl)-L-alanyl]-S-phenylglycine t-butyl ester (DAPT, 10 μM, Sigma) was added 3 times to the described cultures, every other day after final plating^35^,^36^. After final plating cells were grown in N2B27 medium supplemented with 1 mM dibutyryl cAMP (Sigma) from final plating onwards and furthermore supplemented with 10 ng/ml brain derived neurotrophic factor (BDNF) and 10 ng/ml glial derived neurotrophic factor (GDNF, both R&D Systems) after incubation with DAPT.

Cerebral cortex fetal primary human astrocytes (ScienCell™) were cultured and passaged according to the manufacturer’s instructions (in human astrocyte medium, ScienCell™).

### Immunocytochemistry

Cells were fixed for 15 minutes using 4% paraformaldehyde with 4% sucrose in TBS (TrisHCl pH 7.5, NaCl and MilliQ), washed and permeabilized for 15 minutes with Triton-X100 (0.25%) in TBS. After 30 minutes blocking with donkey serum in TBS-Triton (0.25%), cells were incubated overnight at 4 °C with the following antibodies: rabbit or mouse anti-β3 tubulin, rabbit anti-PAX6 (all Covance), mouse anti-HuCHuD, rabbit anti-OCT4, rabbit anti-GS1 (all Thermo Fisher Scientific), chicken anti-MAP2 (Aves), rabbit anti-Nestin, rabbit anti-TBR1, rat anti-CTIP2, mouse anti-SATB2 (all Abcam), rabbit anti-vGLUT1, mouse anti-GAD65 (both Synaptic Systems), mouse anti-S100B (BD transduction laboratories), mouse anti-NANOG, rabbit anti-OTX2, or mouse anti-GFAP (all Millipore). Subsequently, cells were washed and incubated for 1 hour at room temperature with Alexa secondary antibodies (Thermo Fisher Scientific). DAPI was used to counterstain the nuclei. Images were taken either manually with a Leica DMI 4000B microscope or Zeiss LSM 510 (confocal) or automated with the C7000™ High Content Imaging System (confocal, Yokogawa) or Opera Phenix™ High Content Screening System (confocal, Perkin Elmer).

### Live cell calcium imaging

Cells were loaded with 1 μM Fluo-4-AM (Thermo Fisher Scientific) in recording buffer, containing (in mM): CaCl_2_ 1.2; KCl 2.67; NaCl 138; KH_2_PO_4_ 1.47; Na_2_HPO_4_ 8; D-glucose 5.6 (adapted from ref. 55). Cultures were incubated at 37 °C and 5% CO_2_ for 30 minutes and then imaged with an inverted confocal laser scanning microscope (Axiovert 100 M Carl Zeiss, combined with Zeiss LSM510 software) using a Plan-NEOFLUAR 20x objective lens (NA 0.50). 450 frames (61 frames per minute) were recorded per well, of which the first 200 frames represent baseline recordings, followed by 200 frames after acute pharmacological stimulation. Finally, 30 μM glutamate (50 frames) was added to distinguish neurons from non-neuronal cells^56^. Traces of non-neuronal cells, showing only a transient increase in fluorescent intensity upon glutamate addition, were discarded.

A custom-made MATLAB script (based on ref. 16) was used to analyze live cell calcium traces and derive various parameters reflecting characteristics of neuronal activity. In brief, regions of interest (ROIs) were drawn based on time projection images of the recordings. For each ROI traces of fluorescence intensity over time were created and used as substrate for subsequent analyses. Fluorescence traces were normalized to the initial fluorescence intensity (F/F_0_) and average calcium bursting frequency and amplitude were calculated for active cells. Active cells were defined as cells showing at least one peak (i.e. calcium burst) in the fluorescence signal. Fluorescence signals of individual active neurons per well were compared to calculate an average correlation score, indicative of synchronicity of calcium signals (Pearson correlation; -1 up to 1).

To study the influence of voltage-gated sodium channels on calcium oscillations, 100 nM tetrodotoxin was added acutely to cultures after 200 frames. To study glutamatergic and GABAergic contributions to calcium signals and neuronal activity, a combination of 20 μM 6-Cyano-7-nitroquinoxaline-2,3-dione (CNQX, competitive AMPA/kainate receptor antagonist, Sigma) and 50 μM D-(-)-2-Amino-5-phosphonopentanoic acid (D-AP5, competitive NMDA receptor antagonist, Abcam), or 50 μM picrotoxin (GABA_A_ receptor inhibitor, Tocris Bioscience) respectively was added acutely to the cultures after 200 frames. Calcium analysis of these recordings was done by splitting the analysis in two time stretches (before and after the pharmacological intervention), after which calcium parameters from both stretches were compared.

### Electrophysiology – patch clamp

For recording membrane and evoked potentials neurons were cultured on coverslips. In the set-up, coverslips were continuously perfused with extracellular solution containing (in mM) NaCl 125; NaHCO_3_ 25; NaH_2_PO_4_ 1.25; KCl 3; CaCl_2_ 2; MgCl_2_ 1; glucose 25; pyruvic acid 3, pH 7.2–7.4. Extracellular solution was maintained at a temperature of approximately 35°C and bubbled with 95% O_2_, 5% CO_2_. Glass capillaries (Harvard Apparatus) were pulled with a Flaming/Brown micropipette puller (Sutter Instrument) (tip resistance 3–10 MΩ) and filled with intracellular solution containing (in mM) potassium gluconate 135; NaCl 7; HEPES 10; Na_2_ATP 2; Na_2_GTP 0.3; MgCl_2_ 2, pH 7.2–7.4. The voltage clamp technique in whole cell configuration using an EPC10 patch clamp amplifier (HEKA) was performed on cells with documented images. After a cell was successfully patched the resting membrane potential was recorded in zero current clamp mode before starting the experiments. To record action potentials (in current clamp mode), cells were injected with current to maintain the cells at a holding potential of −65 mV. Steps of 50 pA were applied to evoke spiking within a range of −200 to 400 pA. Evoked responses were recorded from 4 time points (week 1: 7–12 days after final plating; week 2: 13–17 days after final plating; week 3: 21–24 days after final plating; week 4–5: 30–36 days after final plating), in order to monitor the progressive maturation of the neuronal culture.

Analysis of these experiments was carried out manually using the Fitmaster software®. Frequencies of action potential firing are reported as the computed mean of the highest frequency observed in each neuron. Depolarizing spikes were considered as action potentials when they were short lasting (shorter than 10 ms at half amplitude) and when the amplitude reached 0 mV. Frequencies of action potentials were calculated as the number of action potentials divided by the current pulse time, also when only one spike was recorded. Rheobase was calculated as the mean of the minimal current injected to evoke an action potential.

Recordings of spontaneous synaptic currents (sPSCs) were made at room temperature using the voltage clamp technique in whole cell configuration using an EPC10 patch clamp amplifier (HEKA). Synaptic activity was sampled at 20 kHz and stored on a PC and subsequently filtered at 1 kHz with a Bessel filter using Patchmaster software (HEKA), running on a PC. Coverslips were placed into petri dishes and fixed on the stage of a Patch Clamp Tower (Luigs and Neumann). An inverted microscope (Olympus IX-50; Luigs and Neumann) was used to observe the cells. Patch pipettes were pulled from borosilicate glass capillaries (outside diameter 1.5 mm, inside diameter 0.87 mm; Hilgenberg) using a horizontal Flaming/Brown micropipette puller (Sutter P-97; Science Products). The pipettes were filled with (in mM) NaCl 10; KCl 120; MgATP 2; HEPES 10; D-glucose 25; GTP 0.1; pH 7.2 with KOH. Spontaneous synaptic activity was recorded in cells maintained in culture for 5 weeks at a holding potential of −80 mV in the presence of extracellular solution containing (in mM) NaCl 141.5; KCl 3; CaCl_2_ 2; HEPES 10; D-glucose 25; pH 7.4 with NaOH.

The number of postsynaptic currents and the number and duration of quasi rhythmic events were analyzed using Clampfit (Molecular Devices). Cells were classified based on their activity pattern. Cells showing no sPSCs at all or up to five single sPCSs per minute were labeled with “no activity”, cells showing 5 or more single events or less than one burst per minute were labeled with “sparse activity” and cells bursting at a frequency higher than one burst per minute were labeled with “frequently bursting”. sPSC bursting frequency was defined as bursts per minute.

### Network morphology

Images of neuronal networks were automatically analyzed using a custom made script (Neuronal Maturation) for Fiji, image processing freeware^57^, which is available upon request. In brief, multidimensional image data sets are read and projected along the Z-axis according to the maximum intensity, after which objects of interest–i.e., nuclei, neurites and synaptic puncta–are detected and quantified.

Nucleus detection is performed by applying an automatic intensity threshold^58^ on DAPI stained images after background subtraction and Gaussian blurring, followed by a watershed-based separation of touching nuclei. To determine the cellular subtype, the mean intensity for each particle was measured in the TBR1, CTIP2, SATB2 and HuC/HuD images. For every neuronal marker an empirical cut-off value was determined, above which cells were considered to belong to a specific neuronal subtype.

To measure neurite outgrowth (MAP2), a multi-tier approach was used, which was based on a dedicated in-house developed analysis for dense neuronal networks, named MorphoNeuroNet^59^. The procedure selectively segments high and low-intensity features in the image and combines them into a single mask.

Synaptic *puncta* (vGLUT1, GAD65) were pre-processed by means of a rolling ball background subtraction, Laplace filtering, automated thresholding^17^,^60^, particle size filtering and counting of the *puncta*. Density of *puncta* was expressed as the number of positive *puncta* per mm^2^ MAP2-positive surface.

### Statistics

Data are shown as mean+ or ±standard error of the mean (SEM). The number of differentiations per experiment refers to the differentiation procedure from hiPSCs to NPCs (either from the same hiPSC line or from a different hiPSC line), while the number of samples refers to the number of wells or cells differentiated towards neurons starting from a single batch of NPCs.

For electrophysiology experiments the differences over time were calculated using one-way ANOVAs considering equal variances. For multiple comparisons between separate time points, Holm-Sidak’s multiple comparisons tests were performed.

For calcium imaging experiments overall differences between groups were calculated using two-way ANOVAs considering equal variances. For multiple comparisons, Dunnett’s *t* tests were performed to detect differences compared to control (for difference between culture conditions: control represents NPCs without co-culture without DAPT; for difference between passage numbers: control represents p0 NPCs in human astrocyte co-cultures) per time point.

## Additional Information

**How to cite this article**: Kuijlaars, J. *et al.* Sustained synchronized neuronal network activity in a human astrocyte co-culture system. *Sci. Rep.* **6**, 36529; doi: 10.1038/srep36529 (2016).

**Publisher’s note:** Springer Nature remains neutral with regard to jurisdictional claims in published maps and institutional affiliations.

## Supplementary Material

Supplementary Information
