# Supplementary material for: Sustained synchronized neuronal network activity in a human astrocyte co-culture system
Source: Sci Rep. 2016 Nov 7;6:36529. doi: 10.1038/srep36529 (PMC5098163; doi:10.1038/srep36529)
Supplement: Supplementary Information [file srep36529-s1.pdf]

Sustained synchronized neuronal network activity in a human astrocyte co-culture system

Jacobine Kuijlaars[a], Tutu Oyelami[b], Annick Diels[b], Jutta Rohrbacher[b], Sofie Versweyveld[b], Giulia Meneghello[b], Marianne Tuefferd[b], Peter Verstraelen[c], Jan R. Detrez[c], Marlies Verschuuren[c], Winnok H. De Vos[c,d], Theo Meert[a,b], Pieter J. Peeters[b], Miroslav Cik[b], Rony Nuydens[b], **Bert Brône[a+]**, **An Verheyen[b+*]**

a. Hasselt University, Biomedical Research Institute, Diepenbeek, B-3590, Belgium
b. Janssen Research & Development, a division of Janssen Pharmaceutica N.V, Beerse, B-2340, Belgium
c. Antwerp University, Department of Veterinary Science, Antwerp, B-2020, Belgium
d. Ghent University, Department of Molecular Biotechnology, Ghent, B-9000, Belgium

[+] Equal contribution

[*] Corresponding author:

An Verheyen, PhD

Janssen R&D

Turnhoutseweg 30

2340 Beerse, Belgium

averhey1@its.jnj.com

Supplementary Figure S1. Primary human astrocytes lose primary cell identity over several passages a) Immunocytochemistry for astrocyte markers GFAP and S100B shows a decrease in marker expression over increasing number of passages of primary human astrocytes. Astrocyte markers are shown in orange and DAPI (nuclei) in blue. b) Micro-array data show a decrease in expression of GFAP and S100B over increasing number of passages of primary human astrocytes. Each datapoint represents 1 biological replicate from the same plate (n = 3).

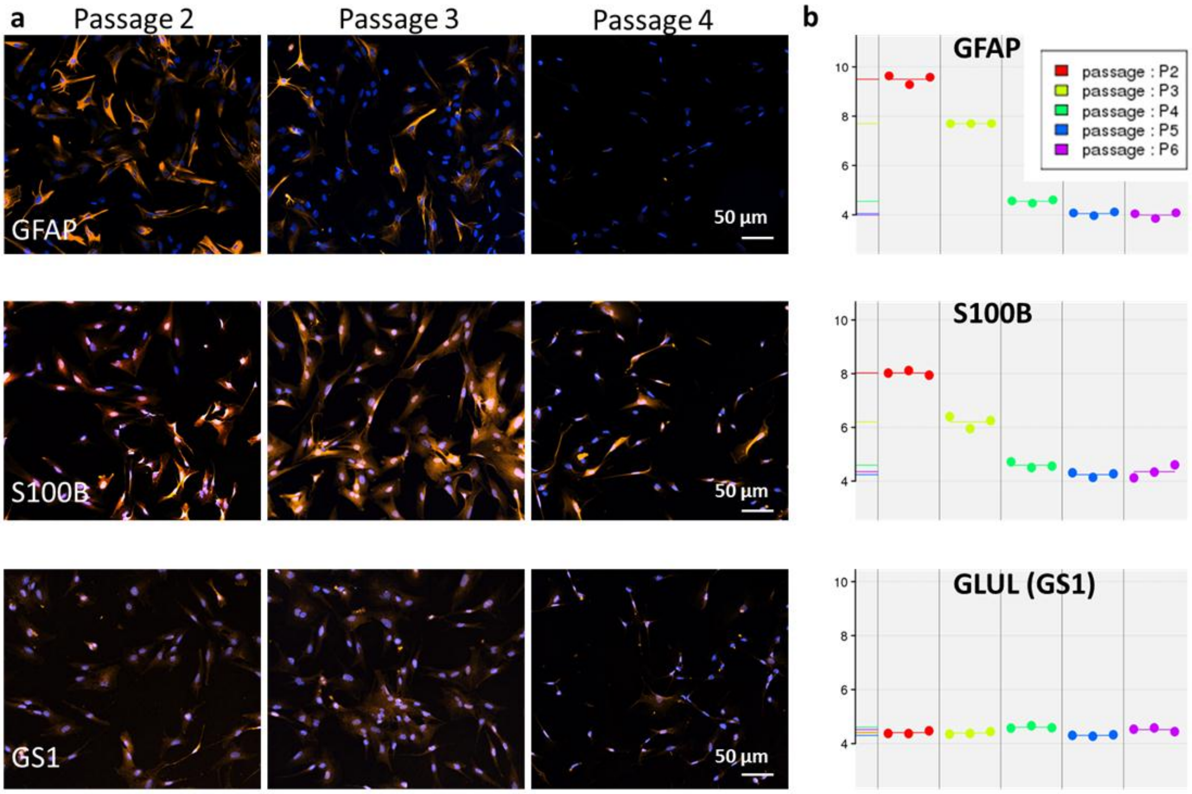

Methods

RNA extraction and microarray analyses

RNA extraction was performed using the RNeasy mini kit (Qiagen). cDNA targets were prepared and labelled using the IVT express kit and then hybridized on Affymetrix® Human Genome U219 array plate in the GeneTitan® instrument

(Affymetrix) according to the manufacturer's protocol. Microarray analysis was performed using the Bioconductor package version 2.12 (working with R version 3.0.1) [1]. Target transcripts of probes were annotated using Entrez Gene based alternative cdf version 15.1.0 [2], assigning probes to 18567 unique transcripts. RMA algorithm was used for pre-processing [3].

References

1 Gentleman, R. C. *et al.* Bioconductor: open software development for computational biology and bioinformatics. *Genome Biol.* **5**, R80 (2004).

2 Dai, M. *et al.* Evolving gene/transcript definitions significantly alter the interpretation of GeneChip data. *Nucleic Acids Res.* **33**, e175-e175 (2005).

3 Irizarry, R. A. *et al.* Exploration, normalization, and summaries of high density oligonucleotide array probe level data. *Biostatistics* **4**, 249-264 (2003).

Supplementary Figure S2. Primary rodent astrocytes induce synchronized neuronal calcium oscillations in human cortical neurons a) hiPSCs differentiated towards cortical neurons in co-culture with primary rat astrocytes are stained with neuronal marker class III β-tubulin (TUBB3), astrocyte marker GFAP and nuclear marker DAPI. b) Co-cultures of hiPSC-derived cortical neurons with primary rat astrocytes (+ DAPT) have been compared to fully human co-cultures and monocultures. Rat co-cultures show an increased percentage of active neurons (Two-way ANOVA, $p < 0.0001$), bursting frequency (Two-way ANOVA, $p < 0.0001$) and synchronization (Two-way ANOVA, $p < 0.0001$) of neuronal calcium oscillations compared to monocultures over a time course of 2 – 5 weeks. When comparing rat and human co-cultures, there is a significant increase in the percentage of active neurons (wk2), bursting frequency (wk2) and correlation score (wk2 and wk3) at early time points, while there is a significant decrease in the amount of active neurons at the latest time point measured (wk 5). The comparison between monocultures and human co-cultures is shown in Figure 3b. $n \geq 3$ from ≥ 2 differentiations, mean ± SEM. * $p<0.05$, ** $p<0.01$, ***$p\leq0.0001$.

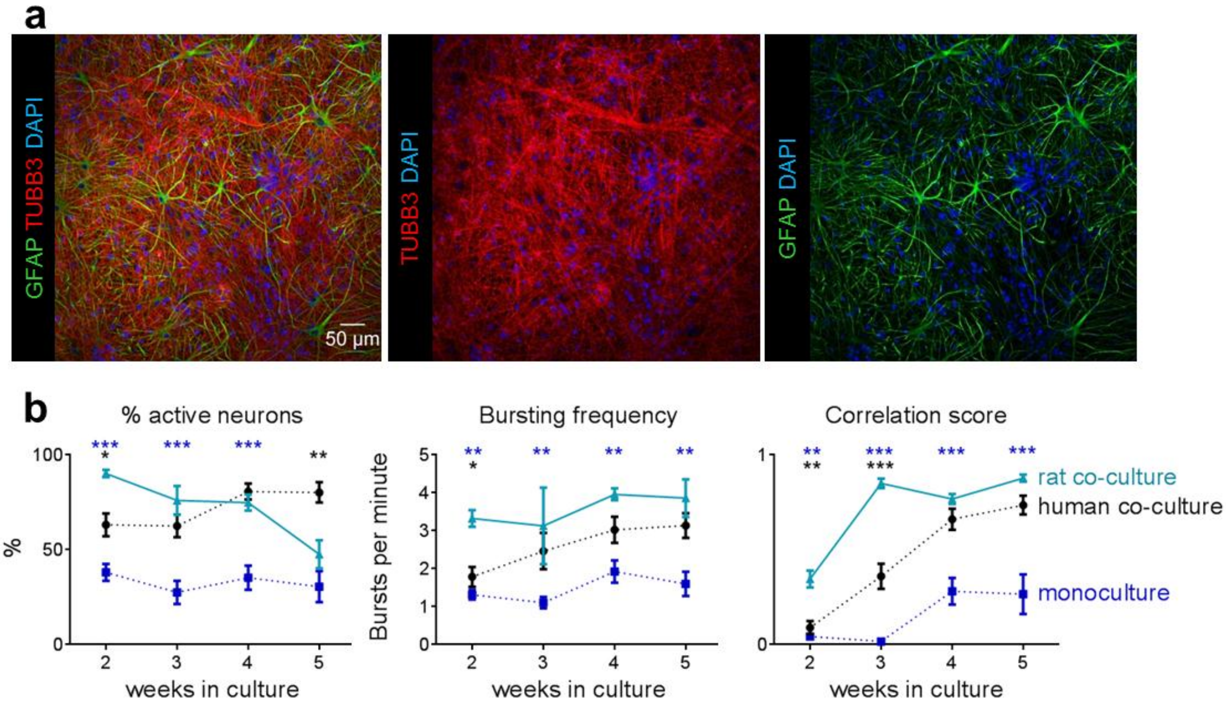

Methods

Cell culture conditions - primary rat astrocytes

Cerebral cortices from E18-19 Wistar rat embryos were dissected in HEPES (7 mM, Sigma) -buffered Hanks Balanced Salt Solution (HBSS) and cells were dissociated enzymatically with trypsin and mechanically triturated through two fire-polished glass pipettes with decreasing diameter. After centrifugation the pellet was resuspended in Minimal Essential Medium (MEM) supplemented with 10% heat-inactivated normal horse serum and 30 mM glucose (Merck) (MEM-horse medium). Cells were grown in cell culture flasks in MEM-horse medium for 12 – 14 days with medium changes every 2 - 3 days. With every medium change loosely attached cells were dislodged and removed. All cell culture supplies were purchased from Thermo Fisher Scientific, unless stated otherwise.

Statistics

For calcium imaging experiments overall differences between groups were calculated using two-way ANOVAs considering equal variances. For multiple comparisons, Dunnett's t tests were performed to detect differences compared to rat co-cultures.
